# Exploring the Therapeutic Potential of Aquaporin-4 Modulation in Sepsis: Inhibitors and Facilitators

**DOI:** 10.3390/ijms27104333

**Published:** 2026-05-13

**Authors:** Alexandru Ionuț Neacșu, Lucian-Ion Giubelan, Bogdan Cătălin, Alexandra Daniela Rotaru-Zăvăleanu, Mădălina Iuliana Mușat, Elena-Mădălina Neniu, Alexandru Ionuț Irimie, Daniel Pirici, Eugen Osiac

**Affiliations:** 1 Doctoral School, University of Medicine and Pharmacy of Craiova, 200349 Craiova, Romania; alex.neacsu1210@gmail.com (A.I.N.);; 2 Infectious Diseases and Pulmonology ‘Victor Babes’ Hospital, Bucharest Road, No.64 (ex 126), 200515 Craiova, Romania; 3 Department of Infectious Diseases, University of Medicine and Pharmacy of Craiova, 200349 Craiova, Romania; 4 Department of Physiology, University of Medicine and Pharmacy of Craiova, 200349 Craiova, Romania; 5 Experimental Research Center for Normal and Pathological Aging, Department of Functional Sciences, University of Medicine and Pharmacy of Craiova, 200349 Craiova, Romania; madalina.musat@umfcv.ro (M.I.M.); eugen.osiac@umfcv.ro (E.O.); 6 Department of Epidemiology, University of Medicine and Pharmacy of Craiova, 200349 Craiova, Romania; 7 Department of Research Methodology, University of Medicine and Pharmacy of Craiova, Petru Rares Street 2, 200349 Craiova, Romania; 8 Department of Histology, University of Medicine and Pharmacy of Craiova, 200349 Craiova, Romania; daniel.pirici@umfcv.ro; 9 Department of Biophysics, University of Medicine and Pharmacy of Craiova, 200349 Craiova, Romania

**Keywords:** sepsis-associated encephalopathy, aquaporin-4, neuroinflammation, astrocytes, blood–brain barrier, neurorepair, polymicrobial sepsis, cecal ligation and puncture, cognitive dysfunction

## Abstract

Sepsis is a life-threatening syndrome driven by a dysregulated host response to infection and is frequently complicated by sepsis-associated encephalopathy (SAE), which contributes to long-term cognitive and neuropsychiatric sequelae. Despite advances in critical care, effective targeted therapies for SAE remain limited. Aquaporin-4 (AQP4), the predominant astrocytic water channel, plays a central role in cerebral water homeostasis, neuroinflammatory signaling, and blood–brain barrier integrity, suggesting its potential involvement in sepsis-induced cerebral dysfunction and neurorepair processes. Polymicrobial sepsis was induced in C57BL/6J mice using the cecal ligation and puncture (CLP) model. AQP4 activity was pharmacologically modulated through either inhibition or facilitation following sepsis induction. Disease severity was assessed using physiological parameters and a modified murine sepsis score. Neurological outcomes were evaluated through standardized behavioral tests assessing locomotor activity, motor coordination, cognitive performance, and depressive-like behavior. Neuroinflammatory and neuronal changes were examined by immunohistochemical analyses of microglial activation (Iba1), astroglial reactivity (GFAP), neuronal integrity (NeuN), and AQP4 expression. Compared with AQP4 facilitation, pharmacological inhibition of AQP4 was associated with a more favorable clinical recovery profile, reflected by lower sepsis severity scores and a more favorable body weight trajectory during the recovery phase. Behavioral analyses demonstrated preserved cognitive function, enhanced motor coordination, and reduced depressive-like behavior in AQP4 inhibitor-treated mice compared with animals receiving AQP4 facilitation. At the histological level, the inhibitor-treated group showed lower microglial and astroglial activation and better preservation of neuronal markers than the facilitator-treated group, whereas AQP4 facilitation exacerbated neuroinflammatory responses and neuronal alterations. These findings highlight a dual, context-dependent role of AQP4 in sepsis-associated cerebral dysfunction. These findings suggest that AQP4 modulation influences sepsis-associated cerebral dysfunction in a context-dependent manner. Within our experimental design, AQP4 facilitation was associated with worse outcomes, whereas AQP4 inhibition was associated with a comparatively more favorable neurobehavioral and histological profile.

## 1. Introduction

Sepsis is a complex, life-threatening syndrome characterized by a dysregulated host response to infection, leading to multi-organ dysfunction and significant mortality worldwide. Recent estimates indicate that sepsis affects approximately 49 million individuals annually, resulting in 11 million deaths and accounting for nearly 20% of global fatalities [1]. Despite advancements in medical care, effective therapeutic strategies remain limited, underscoring the urgent need for novel interventions.

Aquaporins (AQPs) are integral membrane proteins that facilitate water transport across cell membranes, playing crucial roles in maintaining cellular and tissue homeostasis [2,3]. Among the 13 identified AQP isoforms in humans, AQP4 is predominantly expressed in the central nervous system (CNS), particularly in astrocytic end-feet, where it regulates water balance and contributes to the integrity of the blood–brain barrier (BBB) [4,5]. AQP4 inhibition has been suggested as a neuroprotective approach; however, AQP4 facilitation may produce conflicting and possibly harmful effects in the context of systemic inflammation [6].

In the context of sepsis, dysregulation of AQP4 has been implicated in SAE, a severe complication characterized by cognitive impairment and cerebral edema [7]. Experimental studies have demonstrated that during systemic inflammatory responses, such as those observed in sepsis, AQP4 expression is upregulated in the brain, exacerbating vasogenic cerebral edema and contributing to neuronal injury [4]. Conversely, impaired AQP4 function can hinder fluid clearance, leading to the accumulation of interstitial fluid in peripheral organs such as the lungs and kidneys [8]. These findings suggest that modulating AQP4 activity through inhibitors or facilitators could represent a novel therapeutic strategy in sepsis management. AQP4 inhibitors aim to reduce cerebral edema by limiting water influx into the brain parenchyma, while facilitators may enhance water clearance, potentially mitigating peripheral organ dysfunction [4]. Experimental evidence indicates that an acute polymicrobial sepsis episode can induce long-lasting cerebral alterations, including region-specific neuroinflammation, astroglial reactivity, disrupted AQP4 distribution, and enhanced amyloid burden, particularly when sepsis occurs after the onset of amyloid pathology [9]. Animal models, particularly the cecal ligation and puncture (CLP) model in mice, have been instrumental in elucidating the role of AQP4 in sepsis. The CLP model closely mimics the clinical progression of human sepsis, including the systemic inflammatory response and multi-organ dysfunction [10]. Utilizing this model has allowed researchers to investigate the effects of AQP4 modulation on sepsis outcomes, providing valuable insights into potential therapeutic interventions.

Previous studies suggest that AQP4 plays a complex and context-dependent role in central nervous system injury, with effects that may vary according to the type of insult, the timing of modulation, and the subcellular localization of the channel [5,6]. In sepsis-associated encephalopathy, altered AQP4 expression has been linked to cerebral edema, blood–brain barrier dysfunction, astrocytic activation, and cognitive impairment [7,8]. Recent work has also emphasized that, beyond total expression levels, AQP4 polarization and localization at astrocytic endfeet may influence glymphatic clearance, neuroinflammation, and neuronal homeostasis [6]. Taken together, these observations support the need for comparative studies examining the consequences of both AQP4 inhibition and AQP4 facilitation in polymicrobial sepsis [10,11].

Nonetheless, direct experimental comparisons between AQP4 inhibition and facilitation in the realm of polymicrobial sepsis and its related neuroinflammatory consequences are still scarce [11]. In summary, AQP4 plays a significant role in the development of sepsis-related complications, especially within the CNS. Targeting AQP4 through specific inhibitors or facilitators offers a promising therapeutic strategy to alleviate organ dysfunction associated with sepsis. Further research, particularly using established animal models like the CLP model, is essential to advance our understanding and develop effective treatments for this complex syndrome. The aim of this study was to examine the impact of pharmacological modulation of AQP4 activity, utilizing specific inhibitors and facilitators, on neuroinflammatory responses, neuronal integrity, and behavioral outcomes in a murine model of polymicrobial sepsis induced by cecal ligation and puncture.

## 2. Results

### 2.1. Severity of Sepsis

To characterize the septic phenotype induced by CLP in the present experimental setting, physiological and clinical parameters were assessed longitudinally. As expected for polymicrobial sepsis, CLP induced hypothermia, body weight loss, and increased clinical severity, confirming the establishment of a systemic septic phenotype in both treatment groups.

In the present study, AQP4 activity was pharmacologically modulated using TGN-020, an AQP4 inhibitor, and TGN-073, an AQP4 facilitator.

#### Physiological Parameters: Body Temperature, Body Weight, and mMSS

**Body Temperature**

Rectal temperature was assessed at baseline and several post-CLP intervals as a principal indicator of systemic inflammatory response and metabolic dysregulation. After CLP induction, both experimental groups demonstrated an initial decrease in body temperature, aligning with the onset of sepsis-associated hypothermia.

A two-way repeated-measures ANOVA demonstrated a significant main effect of time on body temperature (*p* < 0.0001), indicating dynamic thermoregulatory changes throughout the experimental period. Nonetheless, there was no significant main effect of treatment (AQP4 inhibitor vs. AQP4 facilitator) (*p* = 0.7236), nor was there a significant interaction between time and treatment (*p* = 0.1694). Using Šidák’s correction for post hoc multiple comparisons, we did not find any statistically significant differences between the two groups at any point in time, even on Day 7.

**Figure 1:**
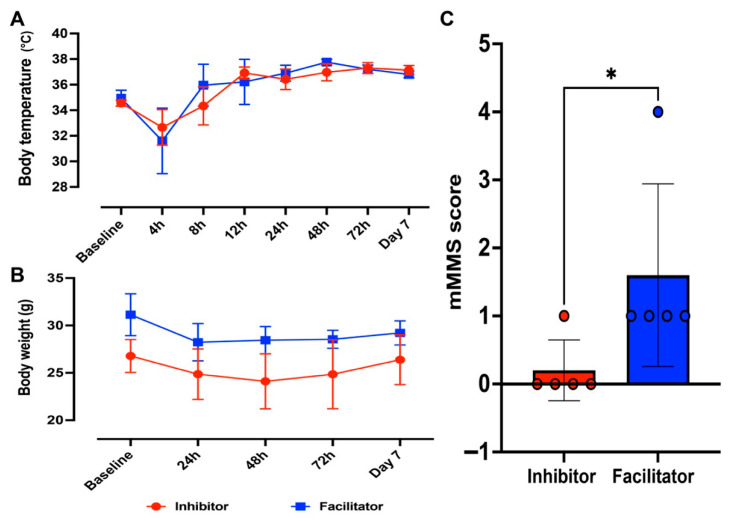
Systemic effects of AQP4 modulation during polymicrobial sepsis. (**A**) Temporal evolution of body temperature following CLP in mice treated with an AQP4 inhibitor or facilitator. Body temperature was measured at baseline and at multiple time points post-CLP. (**B**) Longitudinal body weight changes after CLP induction in animals receiving AQP4 inhibition or facilitation. Because baseline body weight differed between groups, body weight data were interpreted in terms of trajectory over time and recovery relative to baseline rather than as a standalone endpoint comparison at Day 7. (**C**) mMMS assessed at day 7 post-CLP, illustrating overall clinical severity. Statistical analyses included two-way repeated-measures ANOVA for longitudinal parameters and Mann–Whitney U test for mMMS at day 7. *p* < 0.05 was considered statistically significant * *p* < 0.05.

By Day 7 after CLP, body temperatures in both groups were close to normal ranges, with average temperatures of 37.14 ± 0.38 °C in the AQP4 inhibitor group and 36.80 ± 0.31 °C in the AQP4 facilitator group (Figure 1A).

**Body Weight**

Body weight was evaluated longitudinally as a proxy indicator of systemic illness severity, catabolic load, and recovery. Because baseline body weight differed between groups, the Day 7 absolute comparison was interpreted with caution. Here, baseline refers to the pre-treatment body weight measurement. Therefore, the observed between-group difference at baseline was not treatment-induced, but most likely reflects random inter-animal variability after random allocation in a relatively small cohort. Both groups showed weight loss after CLP, followed by partial recovery by Day 7. Inhibitor-treated animals tended to recover closer to their baseline body weight, whereas facilitator-treated animals remained below baseline, indicating different post-sepsis recovery trajectories (Figure 1B). At the final experimental time point (Day 7 post-CLP), mice treated with the AQP4 inhibitor exhibited a mean body weight of 26.38 ± 2.60 g, whereas animals receiving the AQP4 facilitator displayed significantly higher body weights (29.22 ± 1.23 g).

**Modified Murine Sepsis Score (mMSS)**

We used the mMSS to get a more detailed picture of the disease’s severity. This score combines several behavioral and physiological factors to give a full picture of the disease’s impact. At early post-CLP time points (4 h to 72 h), there were no statistically significant differences in mMSS scores between the AQP4 inhibitor and facilitator groups. However, at Day 7 post-CLP, a significant difference emerged between groups. Animals treated with the AQP4 inhibitor showed lower mMSS scores (median: 0.0–1.0), while animals treated with the AQP4 facilitator kept higher scores. The Mann–Whitney U test showed that the mMSS scores in the AQP4 inhibitor group were significantly lower than those in the facilitator group on Day 7 (*p* = 0.0397) (Figure 1C).

### 2.2. Behavioral Assessment

Behavioral performance was assessed to examine the impact of AQP4 modulation on locomotor activity, motor coordination, affective-like behavior, and cognitive function subsequent to sepsis induction. A series of complementary behavioral assessments were utilized, encompassing the OFT, NOR, Beam Walk test, and TST. All tests are done in the same conditions as previously detailed [12,13,14,15].

#### 2.2.1. Open Field Test: Locomotor Activity and Velocity

The OFT was used to measure spontaneous locomotor activity. The total distance traveled and mean velocity were looked at at the beginning, 3 days later, and 7 days later.

**Figure 2:**
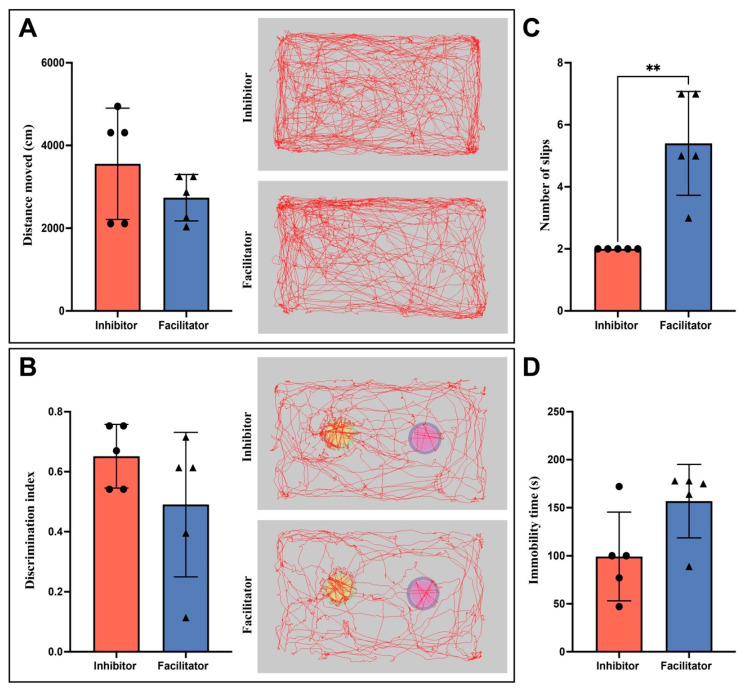
Behavioral assessment at 7 days after CLP. (**A**) OFT showing the total distance traveled (cm) at 7 days after CLP. Representative tracking images depict locomotor trajectories within the open-field arena in inhibitor- and facilitator-treated animals. No significant differences were observed between the inhibitor and facilitator groups (unpaired *t* test with Welch’s correction). (**B**) NOR performance expressed as discrimination index at 7 days after CLP. Representative tracking images depict exploratory trajectories within the testing arena during the test phase. Both groups showed comparable discrimination indices, with no statistically significant difference between inhibitor- and facilitator-treated animals (unpaired *t* test with Welch’s correction). (**C**) Motor coordination assessed by the beam walk test at 7 days after CLP, expressed as the number of slips. Facilitator-treated animals exhibited a significantly higher number of slips compared to the inhibitor group, indicating impaired motor performance (Mann–Whitney test). Data are presented as individual values with central tendency indicated; *n* = 5 animals per group. (**D**) Tail Suspension Test (TST): immobility time at baseline and 7 days post-CLP. No significant difference was observed between groups at baseline. At day 7, facilitator-treated animals showed increased immobility compared with inhibitor-treated animals, without reaching statistical significance; ** *p* < 0.01.

For total distance traveled, a two-way repeated-measures ANOVA indicated a significant main effect of time (*p* = 0.0016), signifying dynamic alterations in locomotor activity throughout the experimental period. A significant main effect of treatment was observed (*p* = 0.0333), indicating overall differences between the AQP4 inhibitor and facilitator groups. Nonetheless, no significant time × treatment interaction was observed (*p* = 0.2155), indicating a similar temporal pattern of change in both groups (Figure 2A).

The analysis of locomotor velocity revealed a significant main effect of time (*p* = 0.0002) and a significant main effect of treatment (*p* = 0.0407). A significant time × treatment interaction was identified (*p* = 0.0212), indicating that the evolution of locomotor velocity over time differed between the two treatment groups. Post hoc Šidák comparisons indicated a significant difference between groups at baseline (*p* = 0.0131); however, no significant differences were noted at 3 or 7 days post-sepsis.

#### 2.2.2. Novel Object Recognition (NOR) Test: Cognitive Performance

We used the NOR test to measure cognitive function. We looked at the percentage of total investigation time and the Discrimination Index (DI).

The analysis of the percentage of total investigation time demonstrated a significant main effect of treatment (*p* = 0.0067), indicating notable differences between groups over time. There was no significant effect of time (*p* = 0.8396) or a significant time × treatment interaction (*p* = 0.0531). Post hoc analysis revealed a significant increase in investigation time in the AQP4 inhibitor group compared to the facilitator group at Day 7 (*p* = 0.0150); however, no significant differences were observed at baseline or Day 3. 

Analysis of the Discrimination Index showed a significant main effect of treatment (*p* = 0.0237), with animals treated with the AQP4 inhibitor having higher DI values. There were no significant effects of time (*p* = 0.6920) or the interaction between time and treatment (*p* = 0.1316). Post hoc Šidák testing revealed a significant difference between groups on Day 3 (*p* = 0.0038), whereas comparisons at baseline and Day 7 did not achieve statistical significance (Figure 2B).

#### 2.2.3. Beam Walk Test: Motor Coordination and Balance

The Beam Walk test was used to measure motor coordination and balance by looking at how long it took to cross the beam and how many times the mouse slipped.

We used two-way repeated-measures ANOVA to look at traversal time. The results showed that treatment had a significant main effect (*p* = 0.0120), which means that there were overall differences between the AQP4 inhibitor and facilitator groups. There was no significant effect of time (*p* = 0.6047) or time × treatment interaction (*p* = 0.3439). Post hoc multiple comparisons failed to reveal significant group differences at specific time points.

Because the slip counts did not follow a normal distribution, Mann–Whitney U tests were used to look at the number of slips at each time point. There were no notable differences between groups at baseline (*p* = 0.1587) or three days post-sepsis (*p* = 0.3968). Conversely, at 7 days post-sepsis, animals administered the AQP4 facilitator demonstrated a markedly greater incidence of slips relative to the inhibitor group (*p* = 0.0079), signifying compromised motor coordination (Figure 2C).

#### 2.2.4. Tail Suspension Test: Depressive-like Behavior

The TST was used to measure behavior that looked like depression. The times for immobility and mobility were looked at at the start of the study and again seven days after sepsis.

At baseline, there were no significant differences observed between treatment groups in either immobility or mobility parameters (all *p* > 0.05). Seven days after sepsis, animals treated with the AQP4 facilitator exhibited heightened immobility and diminished mobility relative to the inhibitor group (Figure 2D); however, these differences did not achieve statistical significance, despite a discernible trend towards modified affective-like behavior (immobility: *p* = 0.0873; mobility: *p* = 0.0794).

#### 2.2.5. Summary of Behavioral Outcomes

Overall, behavioral analyses showed that AQP4 modulation exerted significant effects across multiple functional domains after sepsis. While general movement and motor coordination changed over time in ways that were consistent with systemic illness and recovery, AQP4 inhibition and facilitation had different effects on cognitive performance and fine motor coordination. AQP4 inhibition was correlated with enhanced cognitive performance in the NOR test and diminished motor impairment in the Beam Walk test at later stages, whereas AQP4 facilitation was associated with enduring behavioral deficits.

### 2.3. Neuroinflammatory Response, Neuronal Integrity, and AQP4 Expression in the Cortex and Hippocampus

#### 2.3.1. Microglial Activation (IBA1)

**Figure 3:**
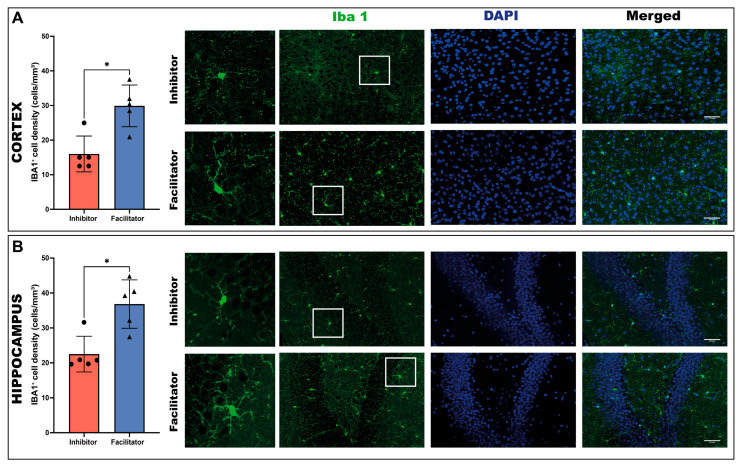
IBA1^+^ microglial cell density in the cortex and hippocampus at day 7. Representative microscopic images show IBA1 immunoreactivity, DAPI nuclear counterstaining, merged images, and higher-magnification inset views of representative microglial cells in the cortex and hippocampus (**A**) Quantification of IBA1-positive microglial cell density in the cortex of animals treated with an AQP4 inhibitor or facilitator. Representative microscopic images show IBA1 immunoreactivity, DAPI nuclear counterstaining, and merged images in the cortex for both experimental groups. (**B**) Quantification of IBA1-positive microglial cell density in the hippocampus of animals treated with an AQP4 inhibitor or facilitator. Representative microscopic images show IBA1 immunoreactivity, DAPI nuclear counterstaining, and merged images in the hippocampus for both experimental groups. Microglia were labeled with IBA1 (green), and nuclei were labeled with DAPI (blue). Data are shown as individual values with median (*n* = 5 animals per group). Normality was assessed using the Shapiro–Wilk test. As data did not meet the assumption of normal distribution, group comparisons were performed using the two-tailed Mann–Whitney U test. A significant increase in IBA1-positive cell density was observed in the facilitator group compared to the inhibitor group in both brain regions (*p* = 0.0159) * *p* < 0.05.

Quantitative analysis of IBA1-immunopositive cells demonstrated substantial region-specific disparities among experimental groups. In the cerebral cortex, the density of IBA1-positive cells was markedly elevated in the facilitator-treated group relative to the inhibitor-treated group (Mann–Whitney test, *p* < 0.05), signifying increased microglial activation in the context of AQP4 facilitation. These results indicate a transition towards an elevated microglial activation state subsequent to AQP4 facilitation (Figure 3A).

A comparable trend was noted in the hippocampus, where facilitator-treated subjects demonstrated a significant elevation in IBA1 immunoreactivity compared to inhibitor-treated subjects (Mann–Whitney test, *p* < 0.05). These results indicate that AQP4 facilitation correlates with an intensified microglial response in both cortical and hippocampal areas (Figure 3B).

#### 2.3.2. Astroglial Reactivity (GFAP)

**Figure 4:**
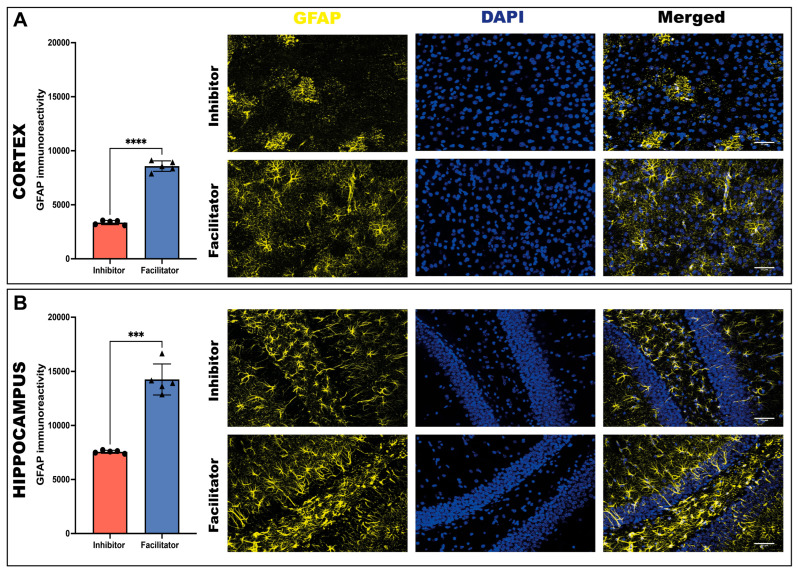
GFAP immunoreactivity in the cortex and hippocampus at day 7. (**A**) Quantification of GFAP signal in the cortex of animals treated with an AQP4 inhibitor or facilitator. Representative microscopic images show GFAP immunoreactivity, DAPI nuclear counterstaining, and merged images in the cortex for both experimental groups. (**B**) Quantification of GFAP signal in the hippocampus of animals treated with an AQP4 inhibitor or facilitator. Representative microscopic images show GFAP immunoreactivity, DAPI nuclear counterstaining, and merged images in the hippocampus for both experimental groups. Astrocytes were labeled with GFAP (yellow), and nuclei were labeled with DAPI (blue). Data are shown as individual values (*n* = 5 animals per group). Normality was assessed using the Shapiro–Wilk test. Group comparisons were performed using a two-tailed unpaired *t* test with Welch’s correction (to account for unequal variances, particularly in the hippocampus). GFAP signal was significantly increased in the facilitator group compared to the inhibitor group in both regions (cortex: *p* < 0.0001; hippocampus: *p* = 0.0004) *** *p* < 0.001; **** *p* < 0.0001.

The evaluation of astrocytic activation through GFAP immunostaining revealed significant disparities between groups. In the cortex, the intensity of GFAP-positive signals was markedly elevated in the facilitator group relative to the inhibitor group (unpaired Welch’s *t* test, *p* < 0.0001), indicating heightened astroglial reactivity (Figure 4A).

GFAP expression was also significantly higher in the hippocampus of animals treated with facilitator (unpaired Welch’s *t* test, *p* < 0.001). The extent of these alterations signifies a robust astrocytic response linked to increased AQP4 activity, especially within hippocampal circuits (Figure 4B).

#### 2.3.3. Neuronal Marker Expression (NeuN)

Quantitative analysis of NeuN immunoreactivity in the cortex at day 7 revealed a significant reduction in the facilitator-treated group compared to the inhibitor-treated group. Statistical analysis using an unpaired *t* test with Welch’s correction showed a highly significant difference between groups (*p* < 0.0001).

**Figure 5:**
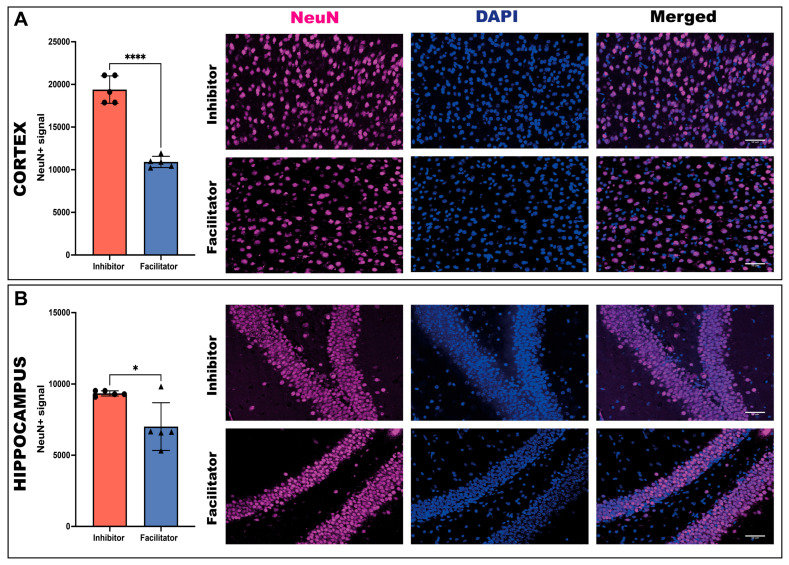
NeuN signal in cortex and hippocampus at day 7. (**A**) Quantification of NeuN signal in the cortex of animals treated with an AQP4 inhibitor or facilitator. Representative microscopic images show NeuN immunoreactivity, DAPI nuclear counterstaining, and merged images in the cortex for both experimental groups. (**B**) Quantification of NeuN signal in the hippocampus of animals treated with an AQP4 inhibitor or facilitator. Representative microscopic images show NeuN immunoreactivity, DAPI nuclear counterstaining, and merged images in the hippocampus for both experimental groups. Neurons were labeled with NeuN (magenta), and nuclei were labeled with DAPI (blue). Data are shown as individual values with mean (*n* = 5 animals per group). Normality was assessed using the Shapiro–Wilk test. As data fulfilled the assumption of normal distribution, group comparisons were performed using a two-tailed unpaired *t* test with Welch’s correction. A significant decrease in NeuN signal was observed in the facilitator group compared to the inhibitor group in both the cortex (*p* < 0.0001) and hippocampus (*p* = 0.0352) * *p* < 0.05; **** *p* < 0.0001.

The mean NeuN signal was markedly lower in the facilitator group compared to the inhibitor group, indicating a substantial decrease in neuronal marker expression. These findings suggest pronounced neuronal alterations in the cortex following treatment with the AQP4 facilitator (Figure 5A).

In the hippocampus, animals treated with the facilitator demonstrated markedly reduced NeuN expression compared to the inhibitor group (unpaired Welch’s *t* test, *p* < 0.05). These findings indicate regionally uniform neuronal modifications linked to AQP4 facilitation (Figure 5B).

#### 2.3.4. AQP4 Expression Analysis

**Mean gray value**

**Figure 6:**
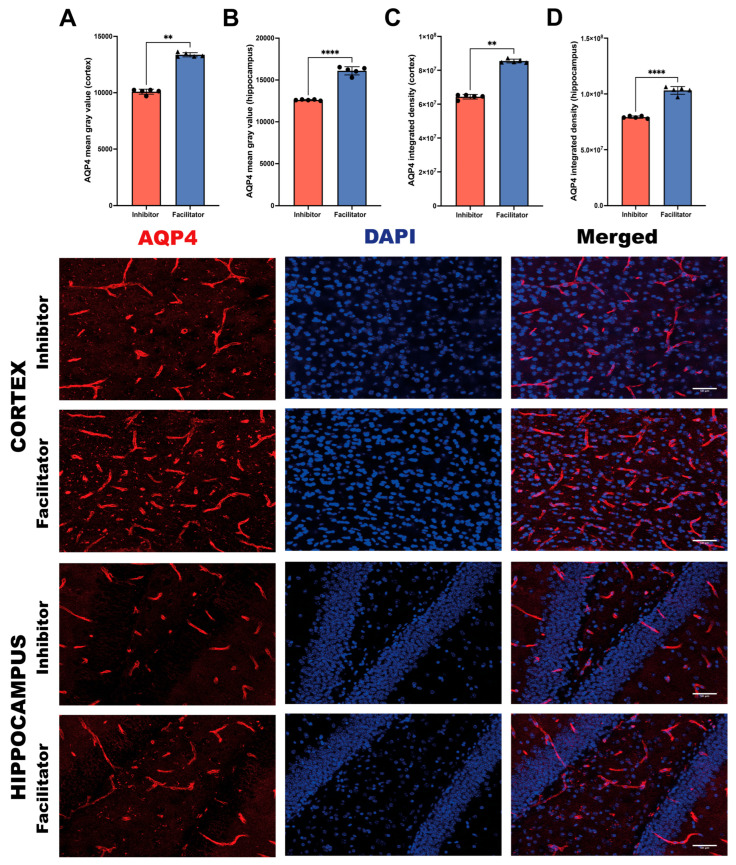
AQP4 expression in the cortex and hippocampus at day 7. (**A**,**B**) Quantification of AQP4 mean gray value in the cortex (**A**) and hippocampus (**B**) in animals treated with an AQP4 inhibitor or facilitator. (**C**,**D**) Quantification of AQP4 integrated density in the cortex (**C**) and hippocampus (**D**). Representative microscopic images show AQP4 immunoreactivity, DAPI nuclear counterstaining, and merged images in the cortex and hippocampus of inhibitor- and facilitator-treated animals. AQP4-positive structures are shown in red, and nuclei are shown in blue. Data are shown as individual values with mean ± SEM or median, as appropriate (*n* = 5 animals per group). Normality was assessed using the Shapiro–Wilk test, and homogeneity of variances was evaluated using an F test. When the assumption of equal variances was violated, group comparisons were performed using a two-tailed unpaired *t* test with Welch’s correction; otherwise, the Mann–Whitney U test was applied. A significant increase in AQP4 expression was observed in the facilitator group compared to the inhibitor group in both brain regions (*p* < 0.05).** *p* < 0.01; **** *p* < 0.0001.

Quantitative analysis of AQP4 mean gray value revealed significant differences between groups. In the cortex, AQP4 mean gray value was significantly increased in the facilitator group compared with the inhibitor group (Mann–Whitney test, *p* < 0.01) (Figure 6A). In the hippocampus, this increase was even more pronounced (unpaired Welch’s *t* test, *p* < 0.0001), indicating enhanced AQP4 expression intensity at the cellular level (Figure 6B).

**Integrated density**

To further evaluate total AQP4 expression, integrated density measurements were conducted. In the cortex, the integrated density of AQP4 was significantly greater in facilitator-treated animals than in inhibitor-treated animals (Mann–Whitney test, *p* < 0.01) (Figure 6C).

In the hippocampus, the integrated density of AQP4 exhibited a pronounced and statistically significant increase in the facilitator group (unpaired Welch’s *t*-test, *p* < 0.0001), indicating a significant upregulation of total AQP4 protein expression in this region (Figure 6D).

#### 2.3.5. Summary of Histopathological Findings

These data collectively indicate that AQP4 facilitation correlates with: increased microglial activation, heightened astrocytic reactivity, modified neuronal marker expression and significant upregulation of AQP4 expression, particularly pronounced in the hippocampus. These region-specific changes show that AQP4 modulation plays a key role in how the body responds to neuroinflammation and keeps neurons healthy during experimental sepsis. Additional supporting information is provided in the Supplementary Materials.

## 3. Discussion

The present study provides significant insights into the role of AQP4 in the pathophysiology of sepsis, emphasizing the contrasting effects of its inhibition and facilitation. Utilizing the CLP model, we demonstrated that AQP4 modulation substantially influences temperature regulation, body weight trends, and overall disease severity as reflected by mMSSs. Although AQP4 facilitation was associated with improved body weight recovery, this apparent systemic benefit did not translate into improved neurological outcomes, as animals displayed increased neuroinflammation, neuronal alterations, and impaired behavioral performance [16]. These findings contribute to a growing body of literature investigating the complex interplay between AQP4 and systemic inflammation, metabolic dysregulation, and cellular homeostasis in sepsis [17,18].

### 3.1. Relationship Between Behavioral Performance and Neuroinflammatory and Neuronal Markers

Behavioral assessment indicated substantial changes in motor coordination, locomotor activity, and affective-like behavior among experimental groups. These functional modifications were assessed concerning the identified histopathological and immunohistochemical changes in the cortex and hippocampus [19,20].

### 3.2. Motor Coordination and Balance

Animals that underwent AQP4 facilitation exhibited markedly compromised motor coordination, as indicated by a higher incidence of slips and extended traversal durations in the beam walk test relative to inhibitor-treated counterparts [21]. These behavioral deficiencies were accompanied by significant IBA1 and GFAP in both cortical and hippocampal areas.

Notably, areas with the highest increases in IBA1 and GFAP immunoreactivity, especially the hippocampus, also had the most severe motor performance problems. This suggests a strong link between neuroinflammatory burden and problems with coordinated motor function [22,23].

### 3.3. Locomotor Activity and Exploratory Behavior

Open field analysis showed that animals treated with a facilitator moved and explored differently. They traveled less distance overall and changed the way they moved. These behavioral modifications corresponded with substantial decreases in NeuN expression in both the cortex and hippocampus, suggesting impaired neuronal integrity or modified neuronal functionality.

The simultaneous occurrence of heightened astrocytic and microglial activation, coupled with diminished neuronal marker expression, substantiates the hypothesis that AQP4 facilitation leads to functional neuronal dysregulation rather than mere inflammatory alterations [24,25].

### 3.4. Affective-like Behavior

The TST for affective-like behavior showed that animals treated with a facilitator were less mobile than those treated with an inhibitor, which suggests that the animals were more depressed [26,27]. These behavioral changes were linked to significant astroglial activation and heightened AQP4 expression, especially in the hippocampus, a region essential for stress response and emotional regulation.

The significant increase in both AQP4 mean gray value and integrated density in the hippocampus indicates that disrupted water homeostasis and astrocytic dysfunction may play a role in the affective behavioral disturbances seen after AQP4 facilitation [28,29].

### 3.5. Integrated Interpretation of Behavioral and Histological Findings

When looked at together, the behavioral problems seen in many functional areas are very similar to neuroinflammatory activation in specific areas, astrocytic reactivity, and changes in the expression of neuronal markers [30]. The hippocampus was identified as a notably susceptible structure, exhibiting significant AQP4 upregulation and strong behavioral associations [31].

These results show that changing the activity of AQP4 is closely related to functional outcomes. For example, increasing AQP4 activity makes neuroinflammation and neuronal dysfunction worse, which leads to measurable problems with motor and emotional behaviors [32]. Collectively, these data endorse an integrative model wherein excessive AQP4 activity exacerbates astrocyte-mediated neuroinflammation, compromises neuronal integrity, and results in enduring behavioral dysfunction subsequent to sepsis [33].

### 3.6. AQP4 Inhibition: A Potential Protective Mechanism

Relative to facilitator-treated animals, the administration of AQP4 inhibitors was associated with a more favorable physiological and clinical profile [34]. Animals in this group exhibited lower mMSSs by Day 7 and a comparatively less severe behavioral and histological profile. These findings are consistent with the possibility that AQP4 inhibition may be associated with attenuation of systemic and neuroinflammatory alterations during sepsis [35].

Mechanistically, AQP4 is implicated in water transport and osmotic balance across cell membranes, particularly in astrocytes and epithelial tissues [28,36]. In the context of sepsis, excessive AQP4 activity may exacerbate tissue edema, disrupt cellular homeostasis, and amplify inflammatory cascades [37]. By inhibiting AQP4, it is plausible that the resultant reduction in tissue edema contributes to improved microcirculatory perfusion, attenuation of hypothermia, and enhanced systemic recovery. Furthermore, reduced inflammatory marker expression and improved histopathological outcomes reported in other studies support the comparatively more favorable profile observed in the inhibitor-treated group [38].

### 3.7. AQP4 Facilitation: Exacerbation of Sepsis Pathophysiology

In contrast, animals treated with AQP4 facilitators experienced worsened outcomes, including sustained hypothermia, profound weight loss, and persistently elevated mMSSs. These findings underscore the potential dangers of enhancing AQP4 activity in the context of systemic inflammation [39,40].

The exacerbation of disease severity observed in this group may be attributable to excessive fluid accumulation and impaired lymphatic drainage, leading to heightened tissue edema and cellular dysfunction [41]. Moreover, the sustained hypothermia and elevated mMSSs suggest that AQP4 facilitation may perpetuate inflammatory signaling and metabolic stress, compounding the systemic insult of sepsis [33,42].

### 3.8. Neuroinflammatory Response: Microglial and Astrocytic Activation

The current results reveal a significant neuroinflammatory response subsequent to polymicrobial sepsis, marked by simultaneous microglial activation and astrocytic reactivity. Increased IBA1 immunoreactivity signifies heightened microglial activation, whereas elevated GFAP expression denotes strong astroglial reactivity in both cortical and hippocampal regions [43]. These changes were particularly pronounced in animals undergoing AQP4 facilitation, while AQP4 inhibition diminished both microglial and astrocytic responses [44].

Considering the preferential localization of AQP4 to astrocytic endfeet [45], these data indicate that astrocyte-mediated mechanisms may be pivotal in enhancing neuroinflammatory signaling, accompanied by secondary microglial activation. Astrocyte–microglia interactions are increasingly acknowledged as pivotal contributors to persistent neuroinflammation in systemic inflammatory disorders, such as sepsis-associated encephalopathy [45,46].

### 3.9. Comparison with Previous Literature

Our findings are consistent with prior research highlighting the dual-edged role of AQP4 in health and disease [47]. While AQP4 is essential for maintaining physiological water homeostasis, its dysregulation has been implicated in various pathological conditions, including cerebral edema, inflammatory diseases, and organ dysfunction during sepsis. Studies in AQP4-knockout models have demonstrated reduced severity of tissue injury and inflammation in experimental sepsis, supporting the therapeutic potential of AQP4 inhibition [48].

Conversely, the deleterious effects of AQP4 facilitation observed in this study align with evidence from models of brain and pulmonary edema, where excessive AQP4 activity exacerbates fluid imbalance and inflammatory responses [17,49]. These parallels suggest that targeted modulation of AQP4, rather than blanket enhancement, is critical for therapeutic success.

### 3.10. Clinical Implications

The differential impact of AQP4 modulation observed in this study has significant translational relevance. The protective effects of AQP4 inhibition highlight its potential as a therapeutic target in managing sepsis and related inflammatory disorders [6,42]. Pharmacological agents designed to selectively inhibit AQP4 could mitigate the systemic and cellular consequences of sepsis, improving patient outcomes [50,51].

However, the findings also caution against strategies that enhance AQP4 activity, which may inadvertently worsen disease severity [19,52]. This underscores the importance of precision medicine approaches that consider the specific pathological context and individual variability in AQP4 expression and activity.

### 3.11. Limitations and Future Directions

While the findings of this study are compelling, several limitations warrant consideration. The sample size, although sufficient to detect significant differences, may limit the generalizability of the results. Additionally, the study focused exclusively on physiological and clinical outcomes; further research is needed to elucidate the molecular and cellular mechanisms underpinning the observed effects of AQP4 modulation. Although sham data were available, they were not included in the main figures, as the primary objective of the study was to compare the relative effects of AQP4 inhibition and facilitation under septic conditions.

An additional limitation is that currently available pharmacological modulators of AQP4 do not offer absolute specificity, and off-target effects cannot be excluded. Therefore, the present results should be interpreted as reflecting pharmacological modulation of AQP4-related pathways rather than definitive proof of a purely AQP4-specific mechanism.

Future studies should also explore the temporal dynamics of AQP4 expression and activity during sepsis progression, as well as the interplay between AQP4 and other key mediators of inflammation and cellular homeostasis. Investigating the effects of AQP4 modulation in diverse experimental models and clinical settings will be critical for validating these findings and advancing therapeutic development.

## 4. Materials and Methods

### 4.1. Animal Model and Ethics Statement

This study utilized C57BL/6J mice as the experimental model to evaluate the role of aquaporin-4 (AQP4) in sepsis. Animals were maintained under standardized laboratory conditions with a controlled temperature of 22 ± 1 °C, a 12-h light/dark cycle, and unrestricted access to food and water. All experimental procedures were conducted in strict accordance with Directive 2010/63/EU for the protection of animals used in scientific research. All procedures performed were in accordance with Directive 2010/63/EU of the European Parliament and the Council, approved by the University Welfare of Experimental Animals committee (225/28.11.2022) and by the Veterinary Sanitary and Food Safety Directorates of Dolj County (17/21.03.2023) according to Romanian and European laws.

### 4.2. Induction of Sepsis: CLP Model

The CLP model, widely recognized as the gold standard for polymicrobial sepsis in preclinical research, was employed to replicate the complex pathophysiology of human sepsis [53,54]. Ten mice were anesthetized with intraperitoneal ketamine (120 mg/kg) and xylazine (10 mg/kg) to ensure adequate analgesia and immobility during the procedure. A midline laparotomy was performed to expose the cecum. Approximately 1 cm from the distal end, the cecum was ligated without obstructing the ileocecal valve to preserve intestinal continuity. A through-and-through perforation was made using a 23-gauge needle, and a small quantity of fecal material was gently extruded to ensure adequate polymicrobial inoculation.

The cecum was repositioned into the peritoneal cavity, and the abdominal incision was closed in two layers with 5-0 sutures. Post-operative fluid resuscitation was achieved with subcutaneous administration of 1 mL of prewarmed sterile saline. Mice were closely monitored at 4-h intervals for clinical signs of sepsis using a modified murine sepsis score (mMSS). The mMSS was calculated as the sum of the individual scores assigned to the following clinical parameters: appearance, level of consciousness/activity, response to stimulus, eye appearance, respiratory rate, and respiratory quality, according to previously published criteria [55,56].

### 4.3. Pharmacological Modulation of AQP4

To explore the therapeutic potential of AQP4 modulation, a total of ten mice subjected to CLP-induced polymicrobial sepsis were randomly assigned to two experimental groups: AQP4 inhibitor-treated (*n =* 5) and AQP4 facilitator-treated (*n* = 5). AQP4 inhibitors (N-(1,3,4-thiadiazol-2-yl)pyridine-3-carboxamide dihydrochloride (TGN-020) (Ukrorgsyntez Ltd., Kiev, Ukraine) were injected intraperitoneally immediately following the CLP procedure at a dose of 100 mg/kg, targeting early-phase cerebral edema [52]. Facilitators (N-(3-(Benzyloxy)pyridin-2-yl) benzene-sulfonamide (TGN-073) (Ukrorgsyntez Ltd., Kiev, Ukraine) were administered a dose of 100 mg/kg. The timing and dosage of pharmacological agents were selected based on previous studies optimizing their therapeutic efficacy in similar models [57].

Both compounds were administered immediately after CLP in order to target the early phase of sepsis when inflammatory and edema-related processes are initiated. The dose of 100 mg/kg was selected based on previously published experimental use of these compounds and to enable direct comparison between AQP4 inhibition and facilitation under identical septic conditions [57].

### 4.4. Behavioral and Physiological Assessments

To comprehensively assess behavioral outcomes, the following tests were conducted: Open Field Test (OFT), Novel Object Recognition (NOR), Beam Walk Test, and Tail Suspension Test (TST). These evaluations provide insights into locomotor activity, anxiety-like behavior, cognitive function, motor coordination, and depressive-like states in rodent models. Behavioral testing and histological analyses were performed by investigators blinded to the experimental groups.

OFT: The OFT is a widely used assay to measure general locomotion and anxiety-related behavior in rodents. Mice were placed individually in an open arena (50 cm (length) × 33 cm (width) × 15 cm (height)) with marked grids. Their movements were recorded for a duration of 10 min using an automated tracking system. Parameters such as total distance traveled, time spent in the center versus periphery, and frequency of rearing were analyzed. Increased time spent in the center is indicative of reduced anxiety-like behavior, while increased locomotion reflects heightened exploratory activity. Mice were placed in an open arena for 10 min, and their movements were recorded using EthoVision XT 17 software (Noldus Information Technology, Leesburg, VA, USA). Parameters measured included total distance traveled, velocity, and time spent in the center zone, indicative of anxiety-like behavior [58].

NOR Test: The NOR test evaluates recognition memory based on rodents’ innate preference for novel objects. The mice were allowed to explore the arena with two identical objects for 6 min. After a retention interval of 1 h, the test phase was conducted by replacing one familiar object with a novel one. Exploration time directed towards each object was recorded, and a discrimination index was calculated to assess memory performance [59].

Beam Walk Test: The Beam Walk Test assesses motor coordination and balance. Mice were trained to traverse a narrow beam (length: 1 m, with a flat surface of 6 mm); two lines were drawn on the beam, the first representing the start line and the second, at a distance of 80 cm, representing the stop line. The beam was elevated 50 cm above a padded surface. The time taken to cross the beam and the number of foot slips were recorded. Improved performance over successive trials indicates intact motor learning and coordination [60].

TST: Depressive-like behavior was evaluated using the tail suspension test. Mice were suspended by the tail with adhesive tape placed approximately 1 cm from the distal end, at a height preventing escape and minimizing distress. The total test duration was 6 min, with the initial 2 min excluded from analysis to allow behavioral stabilization. Immobility duration was recorded and analyzed during the remaining 4 min. Increased immobility time is interpreted as a measure of behavioral despair. This test is widely used for screening potential antidepressant drugs and assessing manipulations expected to affect depression-related behaviors [61].

Physiological parameters, including body weight, rectal temperature, and survival rates, were recorded daily to monitor sepsis progression and therapeutic responses. Baseline body weight and rectal temperature were recorded before administration of the pharmacological compounds. Because animals were randomly allocated and group sizes were small, baseline differences between groups could occur by chance. Rectal temperature was measured under standardized conditions using a lubricated digital rectal probe inserted gently into the rectum.

### 4.5. Tissue Harvesting and Histological Analysis

Seven days post-CLP, mice were subjected to deep anesthesia using ketamine/xylazine and underwent transcardial perfusion with 5 mL saline, succeeded by 4% paraformaldehyde (PFA). The brains were preserved overnight in 4% paraformaldehyde at 4 °C to reduce microglial activation [62]. All immunohistochemistry was performed on 35-μm-thick free-floating coronal brain sections obtained using a vibratome and collected in 0.1 M phosphate-buffered saline (PBS). Free-floating brain sections were permeabilized and blocked in PBS containing 0.5% Triton X-100 and 5% normal serum for 1 h at room temperature, followed by overnight incubation at 4 °C with the following primary antibodies: mouse monoclonal anti-NeuN (Invitrogen, Waltham, MA, USA, MA5-33103; 1:500), goat polyclonal anti-Iba1 (Abcam, Cambridge, UK, ab5076; 1:1000), rabbit polyclonal anti-GFAP (Dako, Glostrup, Denmark, Z0334; 1:1000), and rabbit polyclonal anti-aquaporin-4 antibody (H-80; Santa Cruz Biotechnology, Dallas, TX, USA, catalog no. sc-20812; 1:500). Following washing, sections were incubated for 2 h at room temperature with the corresponding Alexa Fluor-conjugated secondary antibodies: donkey anti-mouse Alexa Fluor 488 (Invitrogen A21202), donkey anti-goat Alexa Fluor 546 (Invitrogen A11056), and donkey anti-rabbit Alexa Fluor 647 (Invitrogen A31573) (all sourced from Invitrogen, Waltham, MA, USA; dilution 1:1000), subsequently accompanied by nuclear counterstaining using DAPI. Histopathological scoring was executed utilizing ImageJ 2.0.0 software (NIH, Bethesda, MD, USA), and quantification was carried out in a blinded fashion to mitigate observer bias [16].

### 4.6. Image Acquisition and Analysis

Images from the cortex and hippocampus were acquired using the 20× objective of an ApoTome Axio Imager.Z2 microscope and Zen software version 2.5 (Carl Zeiss, Jena, Germany). Iba1-positive cells were manually counted using Zen software based on positive immunolabeling and characteristic cellular morphology. Only clearly labeled cells with a well-defined cell body were included in the analysis. The number of Iba1-positive cells was recorded for each field, and cell density was subsequently calculated. NeuN and GFAP immunostaining were quantified using Fiji ImageJ 2.0.0 and Image-Pro Plus 11 software (Media Cybernetics, Bethesda, MD, USA) by assessing marker-related signal intensity under standardized image-processing conditions. Images were converted to grayscale when required, and brightness, contrast, and threshold settings were adjusted uniformly across all samples to optimize signal detection and reduce background interference. Representative images from the cortex and hippocampus were analyzed for each animal, and the values obtained from individual fields were averaged to generate a final mean value for each marker in each brain region. AQP4 expression was quantified in Image-Pro Plus 6.0 using a standardized region of interest (ROI)-based method. For each image, three representative ROIs of identical size were selected, and histogram-derived parameters, including Mean gray value and Integrated density (Sum), were recorded. The mean of the three ROI measurements was calculated for each image, and these values were subsequently averaged to obtain the final representative value for each animal in the cortical and hippocampal regions.

### 4.7. Statistical Analysis

Data were analyzed utilizing GraphPad Prism (version 9.0, GraphPad Software, Boston, MA, USA). Results are presented as mean ± standard deviation (SD). The Shapiro–Wilk test was employed to evaluate data distribution. Parametric data were examined utilizing one-way or two-way repeated-measures ANOVA, succeeded by Šidák’s multiple comparisons test. The Mann–Whitney U test was employed to assess nonparametric data. The threshold for statistical significance was established at *p* < 0.05. In figures, significance levels were denoted as follows: * *p* < 0.05, ** *p* < 0.01, *** *p* < 0.001, and **** *p* < 0.0001. When the assumption of equal variances was violated, group comparisons were performed using a two-tailed unpaired *t* test with Welch’s correction; otherwise, the Mann–Whitney U test was applied.

## 5. Conclusions

This study underscores the important role of AQP4 in modulating sepsis-related neurological and inflammatory outcomes. Within the limits of a two-group comparative design, AQP4 facilitation was associated with greater glial reactivity, lower neuronal marker expression, increased AQP4-related readouts, and a less favorable behavioral profile, whereas inhibitor-treated septic animals showed a comparatively more favorable overall profile. These findings support further investigation of AQP4 as a relevant pathway in sepsis-associated encephalopathy. Future studies should include explicit sham and untreated septic control groups, longitudinal analysis of AQP4 expression and localization, and complementary genetic or mechanistic approaches to distinguish on-target from off-target pharmacological effects and to better define the translational potential of AQP4 modulation in sepsis-related brain dysfunction.

## Data Availability

The raw data supporting the conclusions of this article will be made available by the authors on request.

## Supplementary Materials

The following supporting information can be downloaded at: https://www.mdpi.com/article/10.3390/ijms27104333/s1.

## Abbreviations

The following abbreviations are used in this manuscript:

SAE	sepsis-associated encephalopathy
AQP4	Aquaporin-4
CLP	cecal ligation and puncture
Iba1	microglial activation
GFAP	astroglial reactivity
NeuN	neuronal integrity
AQPs	Aquaporins
CNS	central nervous system
BBB	blood–brain barrier
MSS	modified murine sepsis score
OFT	Open Field Test
NOR	Novel Object Recognition
TST	Tail Suspension Test
PBS	phosphate-buffered saline
PFA	paraformaldehyde
ROI	region of interest
SD	standard deviation
