# Supplementary material for: Exploring the Therapeutic Potential of Aquaporin-4 Modulation in Sepsis: Inhibitors and Facilitators
Source: Int J Mol Sci. 2026 May 13;27(10):4333. doi: 10.3390/ijms27104333 (PMC13207916; doi:10.3390/ijms27104333)
Supplement: Supplementary file 1 [file ijms-27-04333-s001.zip › ijms-4257185-supplementary.pdf]

Supplementary Material: Comparative Evaluation of Experimental and Control Groups

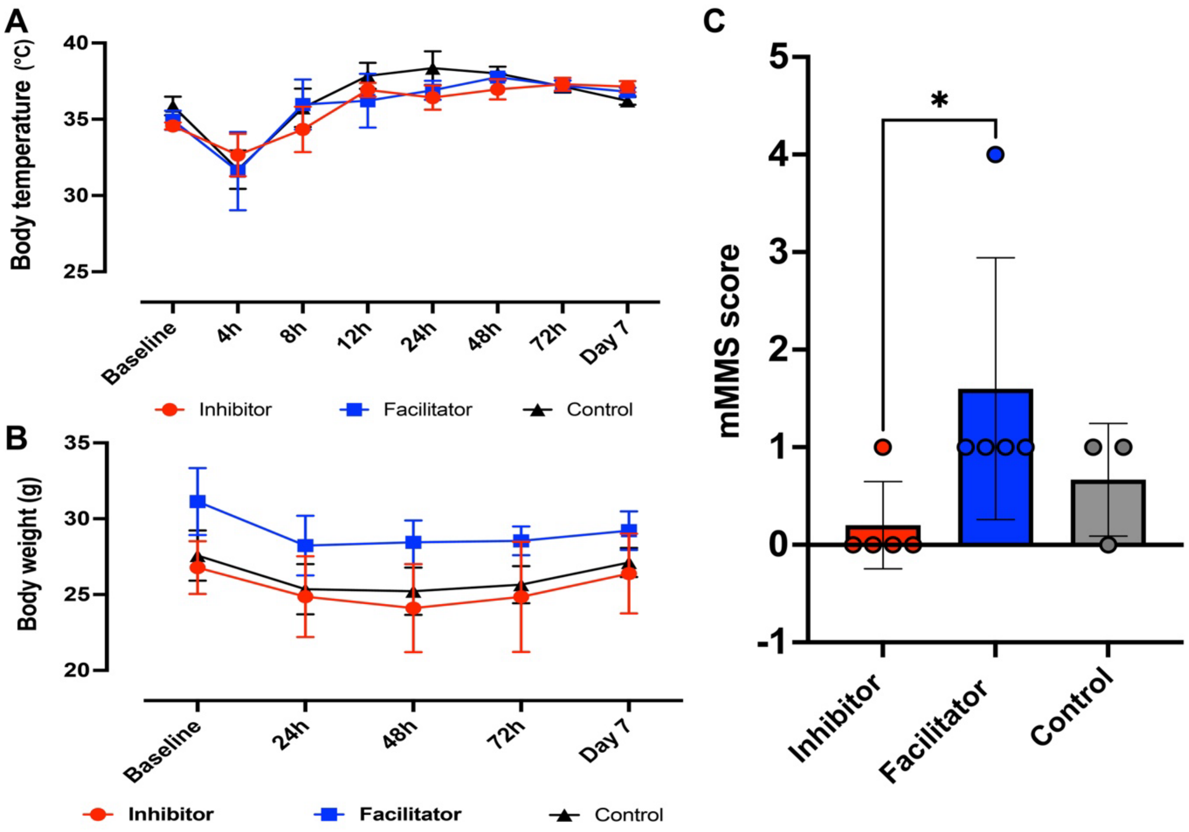

Supplementary material Figure 1. **Systemic effects of AQP4 modulation during polymicrobial sepsis.** (**A**) Temporal profile of body temperature following CLP in mice assigned to the inhibitor, facilitator, and control groups. Body temperature was recorded at baseline and at serial time points after CLP induction. (**B**) Longitudinal changes in body weight after CLP induction in the inhibitor, facilitator, and control groups. Because baseline body weight differed between groups, body weight data were primarily interpreted according to their temporal trajectory and recovery relative to baseline rather than as an isolated endpoint comparison at day 7. (**C**) Modified Murine Sepsis Score (mMSS) assessed at day 7 post-CLP, reflecting overall clinical severity in the three experimental groups. Longitudinal data were analysed using two-way repeated-measures ANOVA. For the day 7 mMSS comparison, an appropriate between-group test was applied. A p value < 0.05 was considered statistically significant.

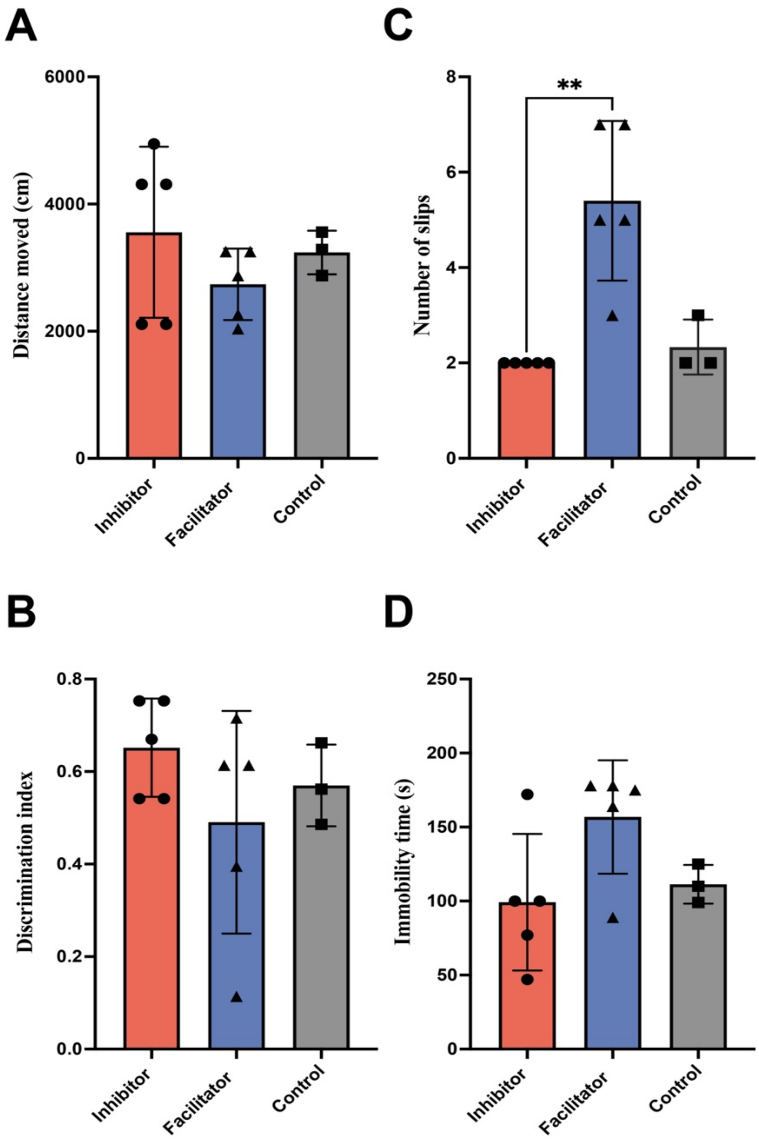

Supplementary material Figure 2. Behavioral assessment at 7 days after CLP in mice assigned to inhibitor, facilitator, and control groups. (**A**) Open field test (OFT), expressed as total distance travelled (cm), at 7 days after CLP. (**B**) Novel object recognition (NOR) performance, expressed as discrimination index, at 7 days after CLP. (**C**) Motor coordination assessed by the Beam walk test, expressed as the number of slips, at 7 days after CLP. (**D**) Tail Suspension Test (TST), expressed as immobility time (s), at 7 days after CLP. Data are presented as individual values overlaid on group summary bars for the inhibitor, facilitator, and control groups. Statistical analyses were performed using tests selected according to data distribution and the number of groups compared. A p value < 0.05 was considered statistically significant.

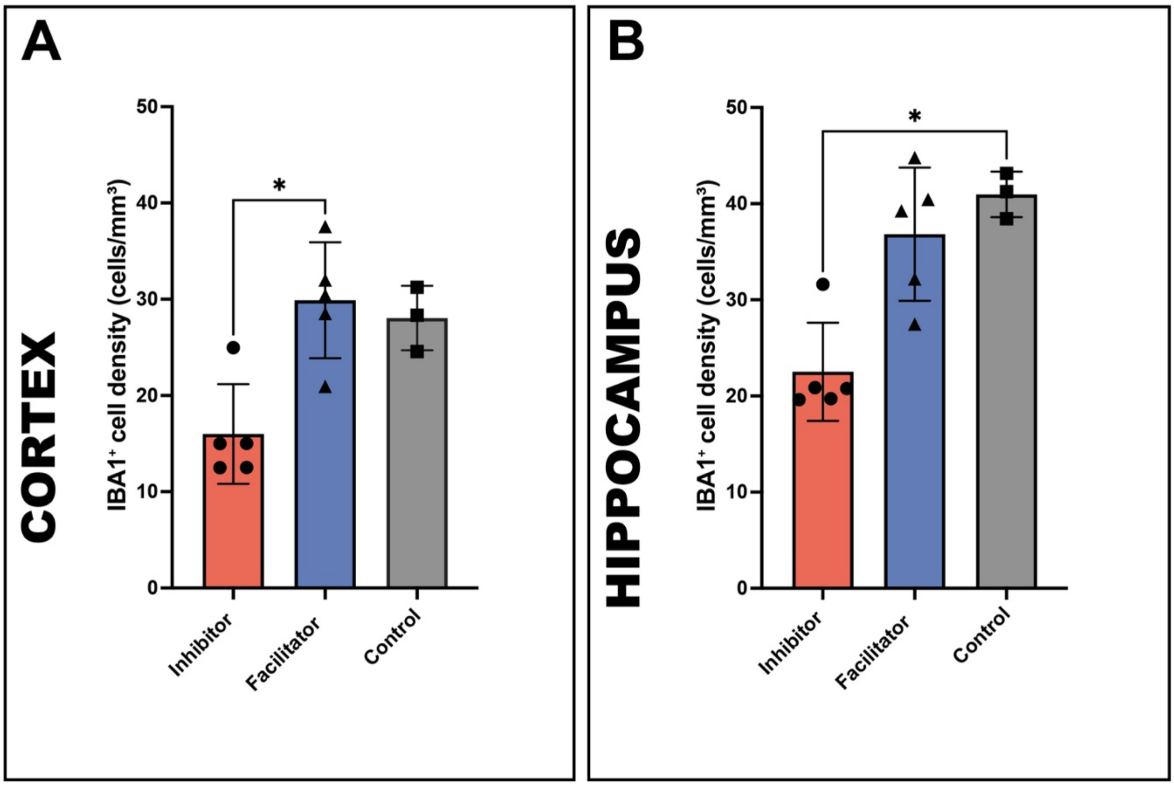

Supplementary material Figure 3. IBA1⁺ microglial cell density in the cortex and hippocampus at day 7 after CLP. (**A**) Quantification of IBA1-positive microglial cell density in the cortex of mice assigned to the inhibitor, facilitator, and control groups. (**B**) Quantification of IBA1-positive microglial cell density in the hippocampus of mice assigned to the inhibitor, facilitator, and control groups. Data are presented as individual values overlaid on group summary bars. Group comparisons were performed using statistical tests selected according to data distribution and the number of groups analyzed. A p value < 0.05 was considered statistically significant.

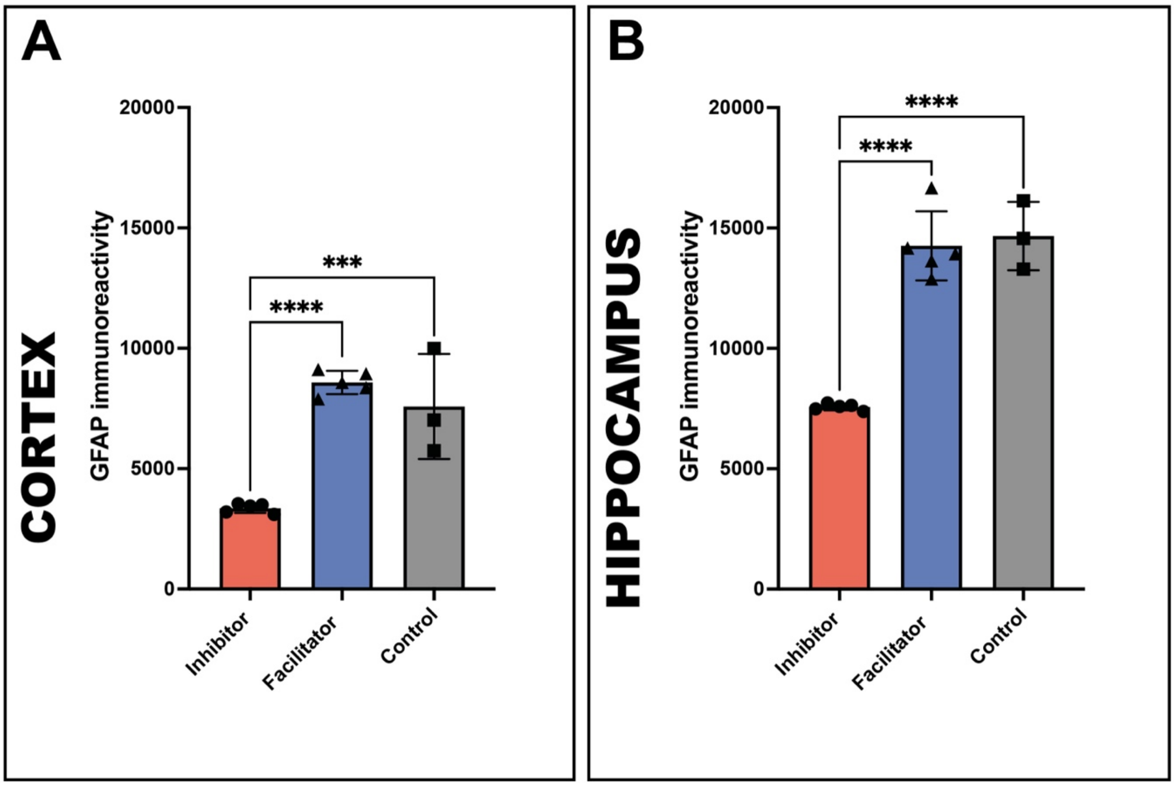

Supplementary material Figure 4. GFAP immunoreactivity in the cortex and hippocampus at day 7 after CLP. (**A**) Quantification of GFAP immunoreactivity in the cortex of mice assigned to the inhibitor, facilitator, and control groups. (**B**) Quantification of GFAP immunoreactivity in the hippocampus of mice assigned to the inhibitor, facilitator, and control groups. Data are presented as individual values overlaid on group summary bars. In the cortex, GFAP immunoreactivity was significantly higher in the facilitator group than in the inhibitor group and was also increased in the control group compared with the inhibitor group. In the hippocampus, both facilitator- and control-treated mice showed significantly higher GFAP immunoreactivity than inhibitor-treated mice. Statistical analysis was performed using tests selected according to data distribution and the number of groups compared. Asterisks indicate the level of statistical significance.

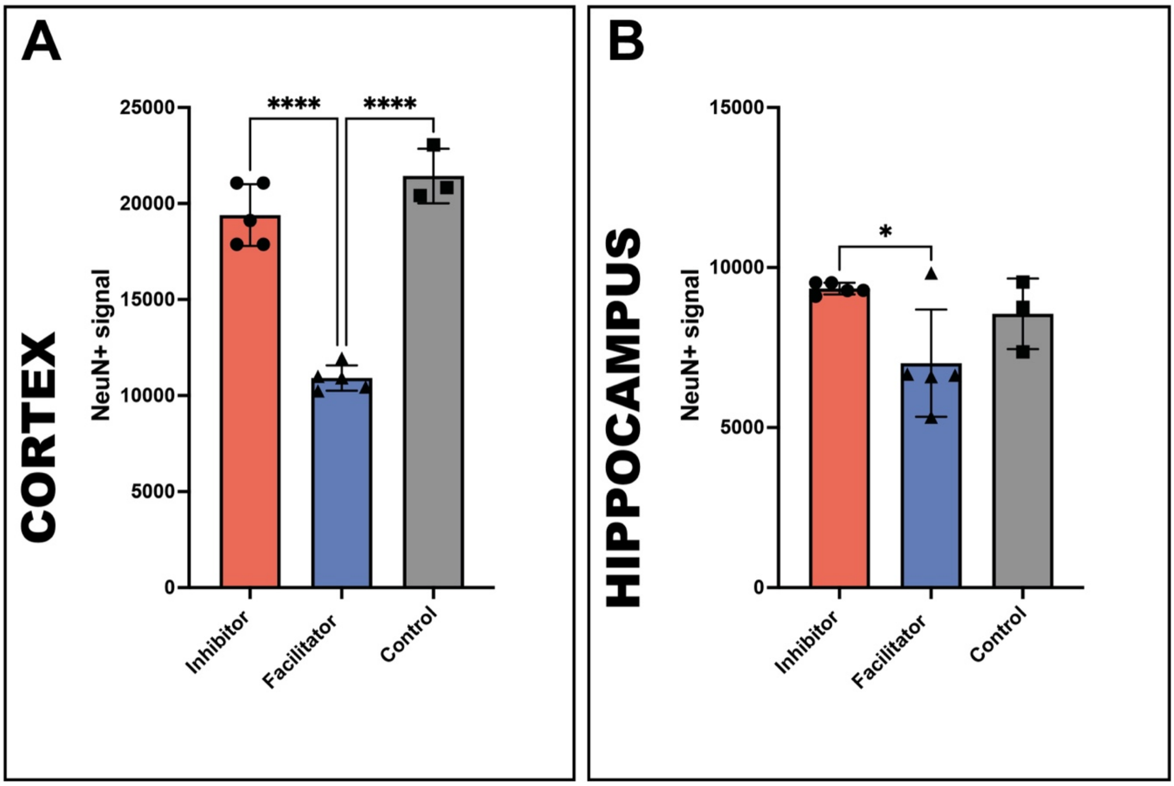

Supplementary material Figure 5. **NeuN signal in the cortex and hippocampus at day 7 after CLP.** (**A**) Quantification of NeuN signal in the cortex of mice assigned to the inhibitor, facilitator, and control groups. (**B**) Quantification of NeuN signal in the hippocampus of mice assigned to the inhibitor, facilitator, and control groups. Data are presented as individual values overlaid on group summary bars. In the cortex, NeuN signal was significantly reduced in the facilitator group compared with both the inhibitor and control groups. In the hippocampus, NeuN signal was significantly lower in the facilitator group than in the inhibitor group. Asterisks indicate the level of statistical significance.

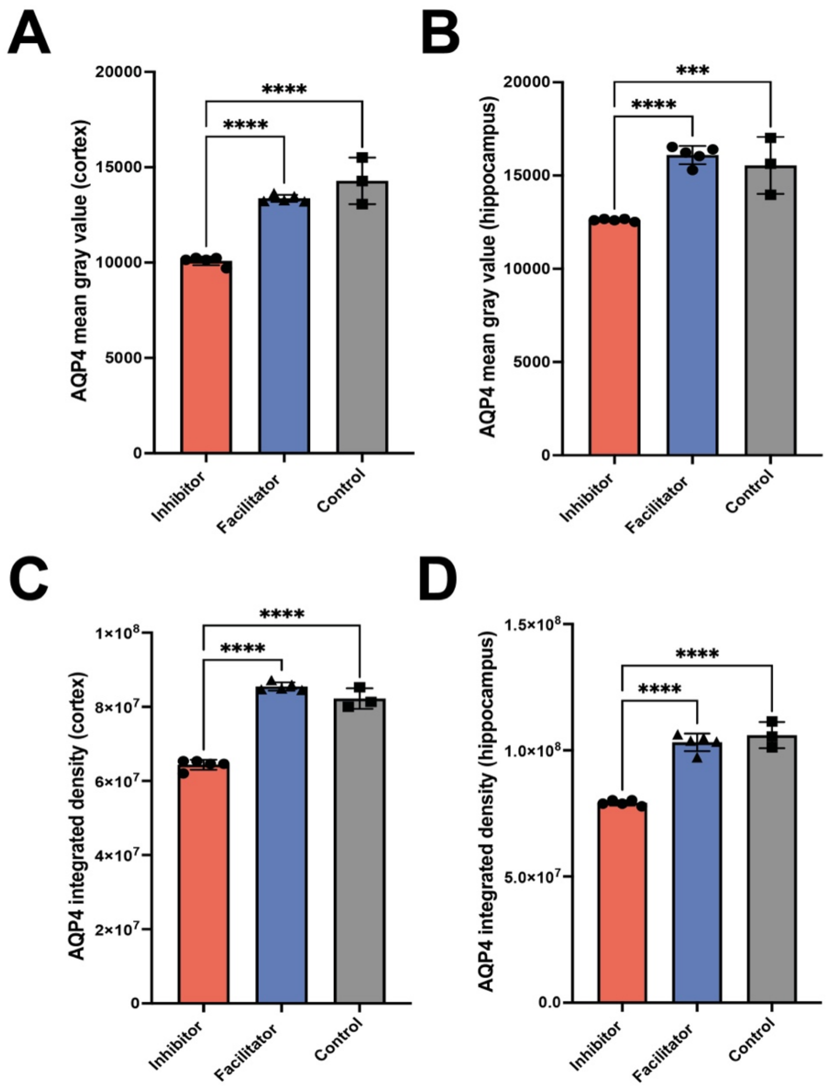

Supplementary material Figure 6. AQP4 expression in the cortex and hippocampus at day 7 after CLP. (**A,B**) Quantification of AQP4 mean gray value in the (**A**) cortex and (**B**) hippocampus of mice assigned to the inhibitor, facilitator, and control groups. (**C,D**) Quantification of AQP4 integrated density in the (**C**) cortex and (D) hippocampus of mice assigned to the inhibitor, facilitator, and control groups. Data are presented as individual values overlaid on group summary bars. In both brain regions, AQP4 mean gray value and integrated density were significantly higher in the facilitator and control groups than in the inhibitor group, as indicated in the figure. Asterisks denote the level of statistical significance.
